# Supplementary material for: Tracing multiple scattering trajectories for deep optical imaging in scattering media
Source: Nat Commun. 2023 Oct 28;14:6871. doi: 10.1038/s41467-023-42525-7 (PMC10613237; doi:10.1038/s41467-023-42525-7)
Supplement: Supplementary file 1 — Supplementary Information [file 41467_2023_42525_MOESM1_ESM.pdf]

Supplementary Information

Tracing multiple scattering trajectories for deep optical imaging in scattering media

Sungsam Kang[1,2], Yongwoo Kwon[1,2], Hojun Lee[1,2], Seho Kim[1,2], Jin Hee Hong[1,2], Seokchan Yoon[1,2,3,*], and Wonshik Choi[1,2,*]

[1]*Center for Molecular Spectroscopy and Dynamics, Institute for Basic Science, Seoul 02841, Korea*

[2]*Department of Physics, Korea University, Seoul 02855, Korea*

[3]*School of Biomedical Convergence Engineering, Pusan National University, Yangsan 50612, Korea*

**Correspondence addressed to* sc.yoon@pusan.ac.kr *and* wonshik@korea.ac.kr

1. Experimental setup for laser scanning reflection-matrix microscopy

1.1 Experimental setup

The laser scanning reflection-matrix microscopy is based on an interferometric confocal reflectance microscope (Fig. S1) [1, 2]. The system utilizes a wavelength-tunable pulsed laser (INSIGHT X3, Spectra physics) whose wavelength is set to 1.3 μm. The repetition rate of the laser is 80 MHz, and its bandwidth is 19 nm, providing a temporal coherence gating window of about 25 μm. The laser beam is split into sample and reference beams at a beam splitter (BS), and then recombined at another BS in front of a camera (InGaAs, Cheetah 800, Xenics, 6.8 kHz framerate). In the sample beam path, a focused beam is delivered to the sample using an objective lens (OL, XLPLN25XWMP2, Olympus, 25x, 1.05 NA), and a galvanometer mirror is used for the raster scanning. The back-scattered beam from the sample is then descanned and delivered to the camera. In the reference beam path, a diffraction grating (DG) is placed at a conjugate plane to the camera for off-axis holographic detection. A scanning mirror (SM) is installed to tune the optical path length of the reference beam. As illustrated in Fig. S1, the reference beam is tilted and collimated at the camera plane with a tilt angle determined by the first-order diffraction angle of DG.

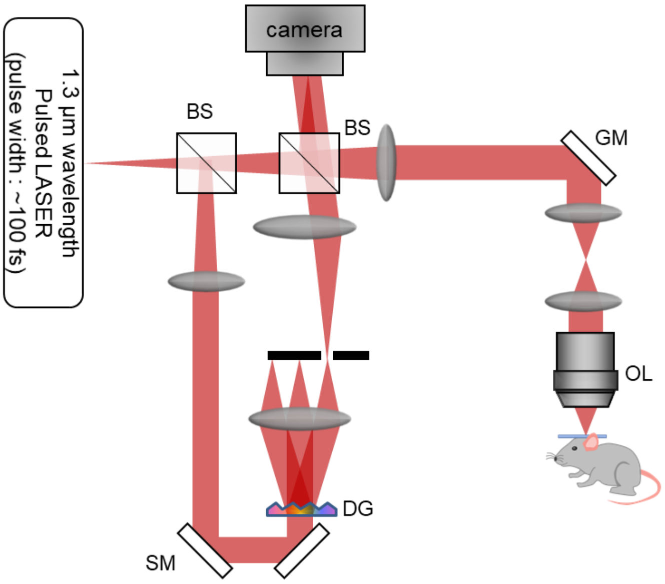

Figure S1. Schematic diagram of the laser scanning reflection matrix microscopy. BS: beam splitter (50:50), GM: galvanometer mirror for raster scanning a focused beam at the sample, SM: scanning mirror to control the optical path length, DG: diffraction grating, and OL: objective lens.

1.2 Construction of a reflection matrix

Figure S2 describes the construction process of a reflection matrix $\boldsymbol{R}$. A mirror sample without a scattering medium was considered as an example. First, descanned interferograms in $(x_{\mathrm{d}}, y_{\mathrm{d}})$-plane are measured by a camera while scanning a focus across the region of interest (ROI). The size of the detection area (region of detection, ROD) was chosen such that the spatially spread reflected wave by the scattering medium is captured. The raw interferogram stack arranged according to the scan index (i) was Fourier transformed to the spatial frequency domain in $\left(k_{x,\mathrm{d}}, k_{y,\mathrm{d}}\right)$-plane. As illustrated, we filtered the AC component of the off-axis interference spectrum (red circle in the upper middle image in Fig. S2) and then recovered the complex maps of the electric field of the reflected wave by inverse Fourier transform. Then, we mapped each descanned complex field map into a laboratory frame in $(x_{\mathrm{o}}, y_{\mathrm{o}})$-plane by laterally shifting it according to its illumination focus position $(x_{\mathrm{in}}, y_{\mathrm{in}})$. Finally, we constructed the reflection matrix $\boldsymbol{R}$ by mapping its columns with vectorized 2D complex field maps in the laboratory frame. As shown in the final image of Fig. S2, column index of $\boldsymbol{R}$ corresponds to the illumination position $(x_{\mathrm{in}}, y_{\mathrm{in}})$ while row index corresponds to $(x_{\mathrm{o}}, y_{\mathrm{o}})$. Since the objective focus is set to the sample plane, $(x_{\mathrm{in}}, y_{\mathrm{in}})$ and $(x_{\mathrm{o}}, y_{\mathrm{o}})$ correspond to the lateral coordinates of the object plane at z_0. Therefore, the recorded matrix $\boldsymbol{R}$ corresponds to $\boldsymbol{R}_{z_0,z_0}$ in the main text.

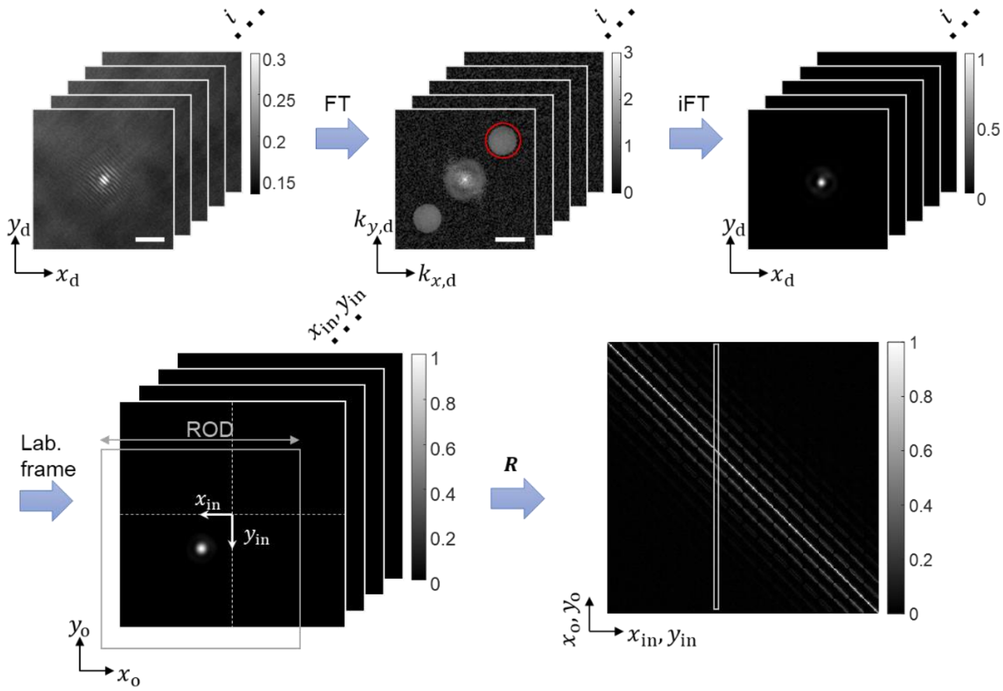

Figure S2. Schematic diagram for the construction of the reflection matrix $\boldsymbol{R}$. Experimentally recorded reflection matrix corresponds to $\boldsymbol{R}_{z_0,z_0}$ in the main text since the objective focus is set to the sample plane.

2. Theoretical model of the MST algorithm

2.1 Multilayer approximation of a thick scattering medium

As illustrated in Fig. S3a, we describe the transmission matrix $\boldsymbol{T}$ of a thick scattering medium as serial production of sub-matrices $\boldsymbol{T}_k$ of thin slabs located at z_k ($k = 1, 2, \ldots, N$): $\boldsymbol{T} = \prod_{k=1}^{N} \boldsymbol{T}_k = \boldsymbol{T}_1 \boldsymbol{T}_2 \cdots \boldsymbol{T}_N$. Note that the production order is arranged such that the input sub-matrix $\boldsymbol{T}_N$ is placed in the rightmost side. If we consider a target object located at z_0, the vectorized incident electric field in the object plane ($(\boldsymbol{\rho}, z_0) = (x, y, z_0)$ plane) can be described as $\mathbf{E}_{\mathrm{in}}(\boldsymbol{\rho}, z_0) = \boldsymbol{T}\mathbf{E}_{\mathrm{in}}(\boldsymbol{\rho}, z_N)$. We can also define a matrix $\boldsymbol{O}$ that describes the object of interest. For a thin depth section, it is a diagonal matrix whose diagonal element $O(\boldsymbol{\rho}, z_0)$ describes the complex amplitude reflectance at each position $(\boldsymbol{\rho}, z_0)$. The electric field reflected by the target object will pass through the same scattering medium, but with opposite propagation direction. As a result, the returning path can be described by the transpose of the input transmission matrix as $\boldsymbol{T}^{\mathrm{T}}$. Combining all these processes, we can model the reflection matrix as

$\boldsymbol{R}^{(\mathrm{M})}_{z_N,z_N} = \boldsymbol{T}^{\mathrm{T}}\boldsymbol{O}\boldsymbol{T}$. In the expression, the subscripts (z_N, z_N) specifies the axial position of the output and input bases, respectively.

In the model, we considered only the forward scattering components by the scattering medium. In general, the experimentally measured reflection matrix also contains multiple scattering components by backward or back-and-forth scattering within the scattering medium, a large fraction of which have no interaction with the object. To attenuate such multiple-scattering components, we apply temporal coherence gating whose time-gating window is set by the round-trip path length of the ballistic waves via the target object. Since the temporal gating has a finite gating window, which is 100 fs or 25 μm in optical path length in our experiment, multiply scattered waves that arrive within the gating window are selectively detected. Due to the narrow time gating window with respect to the width of the total flight time distribution, which is typically on the order of 10 ps or 2.5 mm in optical path length, backward scattering components without interacting with the target object can largely be filtered out [3, 4].

Next, we model the scattering events by individual thin slabs, each of which introduces a phase shift at a plane z_k and the free space propagation by distance to the next slab. The transmission matrix of each thin slab can be described as $\boldsymbol{T}_k = \boldsymbol{P}_{z_{k-1},z_k}\boldsymbol{\Phi}_k$, where $\boldsymbol{\Phi}_k$ is a diagonal matrix whose diagonal element $\mathrm{e}^{i\varphi_k(\boldsymbol{\rho})}$ is phase shift as a function of lateral position $\boldsymbol{\rho}$. $\boldsymbol{P}_{z_{k-1},z_k}$ is a free-space propagation matrix from z_k to z_{k-1} plane. Under this model, the incident electric field at a specific position $(\boldsymbol{\rho}_j, z_k)$ undergoes phase shift $\varphi_k(\boldsymbol{\rho}_j)$ and then propagates in the form of a spherical wave following the Huygens-Fresnel principle of diffraction, as illustrated in Fig. S3b. The transmission matrix $\boldsymbol{T}$ of the scattering medium can be formulated as

$$\boldsymbol{T} = \boldsymbol{T}_1\boldsymbol{T}_2 \cdots \boldsymbol{T}_N = \prod_{k=1}^{N} \boldsymbol{P}_{z_{k-1},z_k}\boldsymbol{\Phi}_k. \tag{S1}$$

Note that the input and output bases of $\boldsymbol{T}$ in the above equation correspond to position vectors in z_N plane and z_0 planes, respectively.

The propagation matrix $\boldsymbol{P}_{z_2,z_1}$ can be described by electric field diffraction of the free space propagation. According to Rayleigh-Sommerfeld diffraction theory [5], the electric field at a point $(\boldsymbol{\rho}_2, z_2)$ from the free-space propagation of the source electric field $E(\boldsymbol{\rho}_1, z_1)$ at z_1 is given by

$$E(\boldsymbol{\rho}_2, z_2) = \int G(\boldsymbol{\rho}_2 - \boldsymbol{\rho}_1; z_2 - z_1) \cdot E(\boldsymbol{\rho}_1, z_1)\mathrm{d}^2\boldsymbol{\rho}_1, \tag{S2}$$

with the Green function given by

$$G(\boldsymbol{\rho}; z) = \frac{1}{i\lambda}\frac{\exp\left(in_0k_0\sqrt{|\boldsymbol{\rho}|^2 + z^2}\right)}{\sqrt{|\boldsymbol{\rho}|^2 + z^2}} \cdot \frac{z}{\sqrt{|\boldsymbol{\rho}|^2 + z^2}}, \tag{S3}$$

where n_0 is the refractive index of the surrounding medium, and $k_0 = 2\pi/\lambda$ is the free-space wave number. In the expression of $G(\boldsymbol{\rho}; z)$, we use a semicolon between $\boldsymbol{\rho}$ and z to distinguish the variable of integration $\boldsymbol{\rho}$ and the distance parameter z. Note that the Green function $G(\boldsymbol{\rho}; z)$ in Eq. (S3) is a form of spherical wave measured in a planar screen perpendicular to z-axis located at a distance z.

Equation (S2) describes the electric field $E(\boldsymbol{\rho}_2, z_2)$ in plane z_2 by a convolution of the Green function $G(\boldsymbol{\rho}; z)$ with the electric field $E(\boldsymbol{\rho}_1, z_1)$ in z_1 plane. Then we can express the convolution integral in Eq. (S2) in the form of matrix-vector multiplication:

$$\mathbf{E}(\boldsymbol{\rho}_2, z_2) = \boldsymbol{P}_{z_2,z_1} \cdot \mathbf{E}(\boldsymbol{\rho}_1, z_1). \tag{S4}$$

The propagation matrix $\boldsymbol{P}_{z_2,z_1}$ takes the Toeplitz form whose $(i, j)^{\mathrm{th}}$ matrix element is given by,

$$[\boldsymbol{P}_{z_2,z_1}]_{ij} = \frac{1}{i\lambda}\frac{(z_2-z_1)\exp\left(in_0k_0\sqrt{\left|\boldsymbol{\rho}_i-\boldsymbol{\rho}_j\right|^2+(z_2-z_1)^2}\right)}{\left|\boldsymbol{\rho}_i-\boldsymbol{\rho}_j\right|^2+z^2}. \tag{S5}$$

The propagation matrix $\boldsymbol{P}_{z_2,z_1}$ in Eq. (S5) describes the diverging spherical wave in free space. Inversely, the back-propagation of electric field with a form of converging spherical wave can be described by conjugate transpose matrix as $\boldsymbol{P}^*_{z_1,z_2} = \boldsymbol{P}^\dagger_{z_2,z_1}$. Therefore, we can easily derive the following properties of $\boldsymbol{P}_{z_2,z_1}$ as,

$$\boldsymbol{P}^\dagger_{z_2,z_1} \cdot \boldsymbol{P}_{z_2,z_1} = \boldsymbol{I}_{z_1,z_1}, \tag{S6}$$

and

$$\boldsymbol{P}_{z_3,z_2} \cdot \boldsymbol{P}_{z_2,z_1} = \boldsymbol{P}_{z_3,z_1}. \tag{S7}$$

In Eq. (S6), $\boldsymbol{I}_{z_1,z_1}$ corresponds to the identity matrix under the input-output position basis laying on the z_1 plane. Finally, we can model the reflection matrix $\boldsymbol{R}^{(\mathrm{M})}_{z_N,z_N}$ in full matrix multiplication with both input and output position bases placed on the entrance of the scattering medium at z_N plane:

$$\boldsymbol{R}^{(M)}_{z_N,z_N} = \left(\prod_{k=N}^{1} \boldsymbol{\Phi}_k \boldsymbol{P}^{\mathrm{T}}_{z_{k-1},z_k}\right) \boldsymbol{O} \left(\prod_{k=1}^{N} \boldsymbol{P}_{z_{k-1},z_k} \boldsymbol{\Phi}_k\right). \tag{S8}$$

So far, we have dealt with the reflection matrix under the input and output position bases laying on z_N plane, the entrance and exit planes. However, in general, we put the target object in the focal plane of the objective lens, and then detect the reflected field at a camera placed in the conjugate plane to the target object. Therefore, the experimentally measured reflection matrix described in the Section 1.2 of this Supplementary Information have the input and output basis in z_0 plane. Therefore, the measured reflection matrix should be modelled in z_0 plane with the application of back-propagation matrices to $\boldsymbol{R}^{(M)}_{z_N,z_N}$:

$$\boldsymbol{R}_{z_0,z_0} = \boldsymbol{P}^*_{z_0,z_N} \boldsymbol{R}^{(M)}_{z_N,z_N} \boldsymbol{P}^*_{z_N,z_0}. \tag{S9}$$

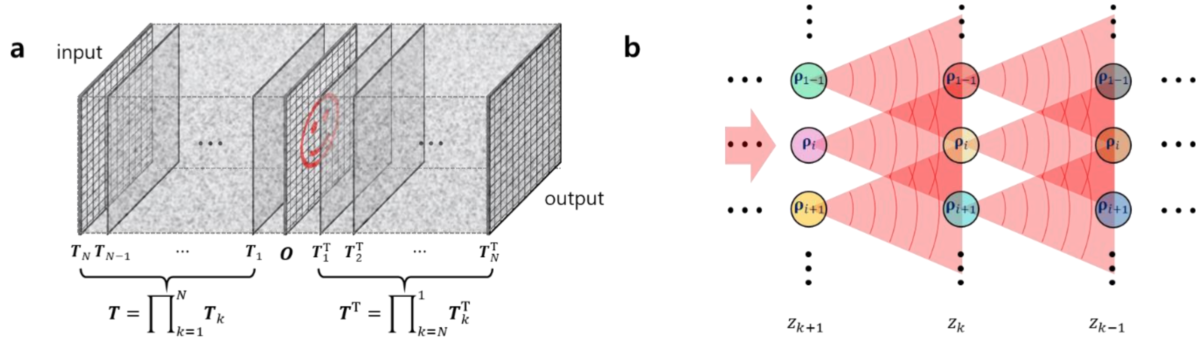

Figure S3. Multi-layer approximation of a thick scattering medium. **a**, Schematic diagram of multi-layer model describing a thick scattering medium. The reflection pathway is unfolded to the right-hand side of the object for the convenience of visualization. **b**, Illustration of optical diffraction between layers.

2.2 Inverse scattering model

In the previous section, we discussed our model for describing the measured reflection matrix $\boldsymbol{R}_{z_0,z_0}$. In the model, we approximated the transmission matrix of the thick scattering medium into finite number of discrete phase plates. If we can find the phase functions φ_k of individual phase plates, then we can obtain the transmission matrix $\boldsymbol{T}$ and the object matrix $\boldsymbol{O}$. Therefore, finding the phase function φ_k is a key part of our MST algorithm. As discussed in the main text, the MST algorithm initially assumes zero phase delay for all phase functions except for a specific plate located at z_k and obtains the phase function φ_k by calculating the wave correlation. Specifically, by propagating the

input side of Eq. (S9) to a specific plate located in z_k, we can obtain the reflection matrix with input basis laying in z_k plane:

$$\begin{aligned}\boldsymbol{R}_{z_0,z_k} &= \boldsymbol{R}_{z_0,z_0}\boldsymbol{P}_{z_0,z_k} = \boldsymbol{P}^*_{z_0,z_N}\boldsymbol{R}^{(M)}_{z_N,z_N}\boldsymbol{P}^*_{z_N,z_0}\boldsymbol{P}_{z_0,z_k} = \boldsymbol{P}^*_{z_0,z_N}\boldsymbol{R}^{(M)}_{z_N,z_N}\boldsymbol{P}^*_{z_N,z_k} \\ &= \boldsymbol{P}^*_{z_0,z_N}\left(\prod_{k'=N}^{1}\boldsymbol{\Phi}_{k'}\boldsymbol{P}^{\mathrm{T}}_{z_{k'-1},z_{k'}}\right)\boldsymbol{O}\left(\prod_{k'=1}^{N}\boldsymbol{P}_{z_{k'-1},z_{k'}}\boldsymbol{\Phi}_{k'}\right)\boldsymbol{P}^*_{z_N,z_k},\end{aligned} \tag{S10}$$

where $\boldsymbol{R}_{z_0,z_k}$ corresponds to the measurement of reflection matrix under the focused illumination at z_k plane while the detection plane is set to the z_0 plane. This corresponds to the defocused illumination to the target object. Now, let's decompose the scattering events in other plates except for z_k plane during the illumination process into the ballistic transmission and the multiple scattering components as follows,

$$\boldsymbol{\Phi}_{k'} = \exp[i\boldsymbol{\varphi}_{k'}(\boldsymbol{\rho})] = \boldsymbol{I}_{z_{k'},z_{k'}} + i\boldsymbol{\varphi}_{k'}(\boldsymbol{\rho}) + \cdots = \boldsymbol{I}_{z_{k'},z_{k'}} + \boldsymbol{M}_{k'} \;\; (\text{for } k' \neq k, \text{ forward}). \tag{S11}$$

Here $\boldsymbol{\varphi}_{k'}(\boldsymbol{\rho})$ is a diagonal matrix whose elements are given by $\varphi_{k'}(\boldsymbol{\rho})$, and $\boldsymbol{M}_{k'}$ is a matrix describing the residual multiple scattering component. In the above equation, we used the power series expansion of the matrix exponential.

Then Eq. (S10) can be simplified as,

$$\begin{aligned}\boldsymbol{R}_{z_0,z_k} &= \boldsymbol{P}^*_{z_0,z_N}\left\{\prod_{k'=N}^{1}\left(\boldsymbol{I}_{z_{k'},z_{k'}} + \boldsymbol{M}_{k'}\right)\boldsymbol{P}^{\mathrm{T}}_{z_{k'-1},z_{k'}}\right\}\boldsymbol{O}\left\{\prod_{k'=1}^{k-1}\boldsymbol{P}_{z_{k'-1},z_{k'}}\left(\boldsymbol{I}_{z_{k'},z_{k'}}\right.\right. \\ &\qquad \left.\left. + \boldsymbol{M}_{k'}\right)\right\}\boldsymbol{P}_{z_{k-1},z_k}\boldsymbol{\Phi}_k\left\{\prod_{k'=k+1}^{N}\boldsymbol{P}_{z_{k'-1},z_{k'}}\left(\boldsymbol{I}_{z_{k'},z_{k'}} + \boldsymbol{M}_{k'}\right)\right\}\boldsymbol{P}^*_{z_N,z_k} \\ &= \boldsymbol{P}^*_{z_0,z_N}\left\{\left(\boldsymbol{P}_{z_0,z_N}\right)^{\mathrm{T}} + \boldsymbol{M}^{(\text{out})}_{z_N,z_0}\right\}\boldsymbol{O}\left\{\boldsymbol{P}_{z_0,z_{k-1}} + \boldsymbol{M}^{(\text{in})}_{z_0,z_{k-1}}\right\}\boldsymbol{P}_{z_{k-1},z_k}\boldsymbol{\Phi}_k\left\{\boldsymbol{P}_{z_k,z_N}\right. \\ &\qquad \left. + \boldsymbol{M}^{(\text{in})}_{z_k,z_N}\right\}\boldsymbol{P}^*_{z_N,z_k} \\ &= \boldsymbol{O}\boldsymbol{P}_{z_0,z_k}\boldsymbol{\Phi}_k + \boldsymbol{M}\end{aligned} \tag{S12}$$

Here $\boldsymbol{M}^{(\text{in,out})}_{z_j,z_i}$ is the residual multiple scattering from the layer at z_i to z_j for input and output process, respectively, and $\boldsymbol{M}$ is overall residual multiple scattering. Note that we also set $\boldsymbol{\Phi}_k = \boldsymbol{I}_{z_k,z_k} + \boldsymbol{M}_k$ for the returning path. Equation (S12) is the Eq. (2) in the main text.

As shown in the first term of Eq. (S12), $\boldsymbol{\Phi}_k$ is factorized in the rightmost side. Since $\boldsymbol{\Phi}_k$ and $\boldsymbol{O}$ are diagonal matrices, a specific column vector for a coordinate $(\boldsymbol{\rho}_1, z_k)$ in Eq. (S12) can be written as

$$\begin{aligned}\boldsymbol{R}_{z_0,z_k}(\boldsymbol{\rho};\boldsymbol{\rho}_1) &= E(\boldsymbol{\rho}, z_0; \boldsymbol{\rho}_1, z_k) \\ &= \mathrm{e}^{i\varphi_k(\boldsymbol{\rho}_1)}O(\boldsymbol{\rho}, z_0)G(\boldsymbol{\rho}-\boldsymbol{\rho}_1; z_0 - z_k) + \boldsymbol{M}(\boldsymbol{\rho};\boldsymbol{\rho}_1).\end{aligned} \tag{S13}$$

Therefore, the inner product of two arbitrary column vectors of $\boldsymbol{R}_{z_0,z_k}$ after normalizing with the Green function can be written as

$$\left\langle\frac{\boldsymbol{R}_{z_0,z_k}(\boldsymbol{\rho};\boldsymbol{\rho}_1)}{G(\boldsymbol{\rho}-\boldsymbol{\rho}_1; z_0 - z_k)}, \frac{\boldsymbol{R}_{z_0,z_k}(\boldsymbol{\rho};\boldsymbol{\rho}_2)}{G(\boldsymbol{\rho}-\boldsymbol{\rho}_2; z_0 - z_k)}\right\rangle_{\boldsymbol{\rho}} = \mathrm{e}^{i[\varphi_k(\boldsymbol{\rho}_1)-\varphi_k(\boldsymbol{\rho}_2)]}\sum_{\boldsymbol{\rho}}|O(\boldsymbol{\rho}, z_0)|^2 + M', \tag{S14}$$

where M' represents the sum of inner product between $\boldsymbol{M}(\boldsymbol{\rho};\boldsymbol{\rho}_1)$ and $\boldsymbol{M}(\boldsymbol{\rho};\boldsymbol{\rho}_2)$ after normalizing the Green function as well as cross terms generated by inner product. Finally, we can obtain the first approximate values of the phase function φ_k as,

$$\varphi_k^{(1)}(\boldsymbol{\rho}) = \arg\left\langle\frac{\boldsymbol{R}_{z_0,z_k}(\boldsymbol{\rho};\boldsymbol{\rho})}{G(\boldsymbol{\rho}-\boldsymbol{\rho}_1; z_0 - z_k)}, \frac{\boldsymbol{R}_{z_0,z_k}(\boldsymbol{\rho};\boldsymbol{0})}{G(\boldsymbol{\rho}-\boldsymbol{\rho}_2; z_0 - z_k)}\right\rangle_{\boldsymbol{\rho}} = \varphi_k(\boldsymbol{\rho}) + \delta\varphi_k^{(1)}(\boldsymbol{\rho}) \tag{S15}$$

with a phase error $\delta\varphi_k^{(1)}$. Here we assumed $\boldsymbol{\rho}_1 = \boldsymbol{\rho}$, and $\boldsymbol{\rho}_2 = \boldsymbol{0}$, and $\varphi_k(\boldsymbol{0}) = 0$ without loss of generality.

Although we described the phase quantification process in Eq. (S15) as if we compare only two column vectors of propagated reflection matrix $\boldsymbol{R}_{z_0,z_k}$, the actual algorithm used in our study utilizes the whole column vectors to determine the phase function with substantially enhanced the fidelity of convergence. More specifically, we define a Green's function normalized matrix $\boldsymbol{S}_{z_0,z_k}$ whose matrix elements are given by

$$\boldsymbol{S}_{z_0,z_k}(\boldsymbol{\rho}, z_0; \boldsymbol{\rho}', z_k) \equiv \frac{E(\boldsymbol{\rho}, z_0; \boldsymbol{\rho}', z_k)}{G(\boldsymbol{\rho}-\boldsymbol{\rho}'; z_0 - z_k)} = \mathrm{e}^{i\varphi_k(\boldsymbol{\rho}')} O(\boldsymbol{\rho}, z_0) + \boldsymbol{M}'(\boldsymbol{\rho}; \boldsymbol{\rho}'). \tag{S16}$$

Then we quantify the phase function of kth phase plate by solving

$$\arg\min_{\boldsymbol{\varphi}_k^{(1)}} \left\| \boldsymbol{S}_{z_0,z_k} - \tau \boldsymbol{o}^{\mathrm{T}} \times \mathrm{e}^{i\boldsymbol{\varphi}_k^{(1)}} \right\|_2, \tag{S17}$$

where $\boldsymbol{o}^{\mathrm{T}}$ is a transpose of the vectorized object function (column vector), $\mathrm{e}^{i\boldsymbol{\varphi}_k^{(1)}}$ is vectorized phase function of the corresponding phase plate (raw vector), and $\|\boldsymbol{A}\|_2$ implies the Frobenius norm of a matrix $\boldsymbol{A}$. This minimization process is identical to finding the first singular vector of $\boldsymbol{S}_{z_0,z_k}$, and we utilized the well-known power iteration method [6].

Despite the more robust phase quantification process in Eq. (S17), the phase error $\delta\varphi_k^{(1)}$ is not negligible when the contribution of the multiple scattering by the other layers at $z_{k'} (k' \neq k)$ is significant. For this reason, we use a superscript (1) to specify that the phase function in Eqs. (S15)-(S17) is the first approximation. As discussed in the Method section of the main text, $\varphi_k^{(1)}(\boldsymbol{\rho})$ is asymptotically close to $\varphi_k(\boldsymbol{\rho})$. Therefore, compensating the first approximation of the phase function by multiplying a diagonal matrix $\mathrm{e}^{-i\boldsymbol{\varphi}_k^{(1)}(\boldsymbol{\rho})}$ to the right side of $\boldsymbol{R}_{z_0,z_k}$ will reduce the amount of multiple scattering by the layer k to some extent. Next, we perform the same operation for all the other layers in sequence.

In addition, we also account for the scattering layers in the output pathway by repeating the previous procedures with $\boldsymbol{R}_{z_0,z_0}^{\mathrm{T}}$. In principle, the phase function of each layer for the returning path from the target object is identical to that in the illumination path due to the reciprocity of reflection imaging geometry. However, due to the imperfection of the experiment, they can be different to a certain extent and thus needs to be processed separately.

Figure S4 describes the flowchart of the iteration process. A series of blue boxes in Fig. S4 indicate the finding of phase functions from layer N to layer 1 in the lth iteration step. For the efficiency of computation, the outmost layer in z_N is identified in its input and output paths, as illustrated in the first blue boxes in Fig. S4. We define an intermediate matrix $\boldsymbol{R}'_{z_k,z_k} \equiv \boldsymbol{P}_{z_k,z_0} \boldsymbol{R}_{z_0,z_0} \boldsymbol{P}_{z_0,z_k}$ to reduce the required number of matrix multiplications. With this intermediate matrix, we need only two matrix multiplications for each input-output iteration step. Here $\boldsymbol{R}_{z_0,z_0}^{(k,l)}$ denotes the reflection matrix corrected for the layer k in the lth iteration. In the first blue box, the lth iteration begins with $\boldsymbol{R}_{z_0,z_0}^{(1,l-1)}$, which is the correction of the layer 1 in the (l-1)[th] iteration, and returns $\boldsymbol{R}_{z_0,z_0}^{(N,l)}$, the correction of layer N in the lth iteration. In the second blue box, we perform the same procedure for the layer N-1 starting from the previously corrected reflection matrix $\boldsymbol{R}_{z_0,z_0}^{(N,l)}$. This step goes on to layer 1. Once the input-output iteration is finished for all the layers, then we repeat the whole process as indicated by the blue arrow in Fig. S4. If we set the number of iterations for the input-output iteration N_{io}, the total number of layers N, and the number of the whole iteration step N_{tot}, then the total number of required iterations becomes $N_{\mathrm{io}} N N_{\mathrm{tot}}$ with $2(N_{\mathrm{io}} + 2) N N_{\mathrm{tot}}$ matrix multiplications.

After the entire iteration process is finished, we can obtain the final solution of the phase function $\varphi_k^{\mathrm{c}}(\boldsymbol{\rho})$ by summation of the phase retardations during the iteration process, i.e., $\varphi_k^{\mathrm{c}}(\boldsymbol{\rho}) = \sum_n \varphi_k^{(n)}(\boldsymbol{\rho})$. Next, we can construct the correction transmission matrix $\boldsymbol{T}^{\mathrm{c}}$ from $\varphi_k^{\mathrm{c}}(\boldsymbol{\rho})$ by Eq. (S1). By multiplying the inverses of $\boldsymbol{T}^{\mathrm{c}}$ and $(\boldsymbol{T}^{\mathrm{c}})^{\mathrm{T}}$ on the input and output sides of the measured reflection matrix $\boldsymbol{R}_{z_N,z_N}$,

respectively, we obtain a corrected reflection matrix, $\boldsymbol{R}^{\mathrm{c}}_{z_0,z_0} = [(\boldsymbol{T}^{\mathrm{c}})^{\mathrm{T}}]^{-1}\boldsymbol{R}_{z_N,z_N}(\boldsymbol{T}^{\mathrm{c}})^{-1}$. We then obtain the object function $O(\boldsymbol{\rho}, z_0)$ from the diagonal elements of $\boldsymbol{R}^{\mathrm{c}}_{z_0,z_0}$.

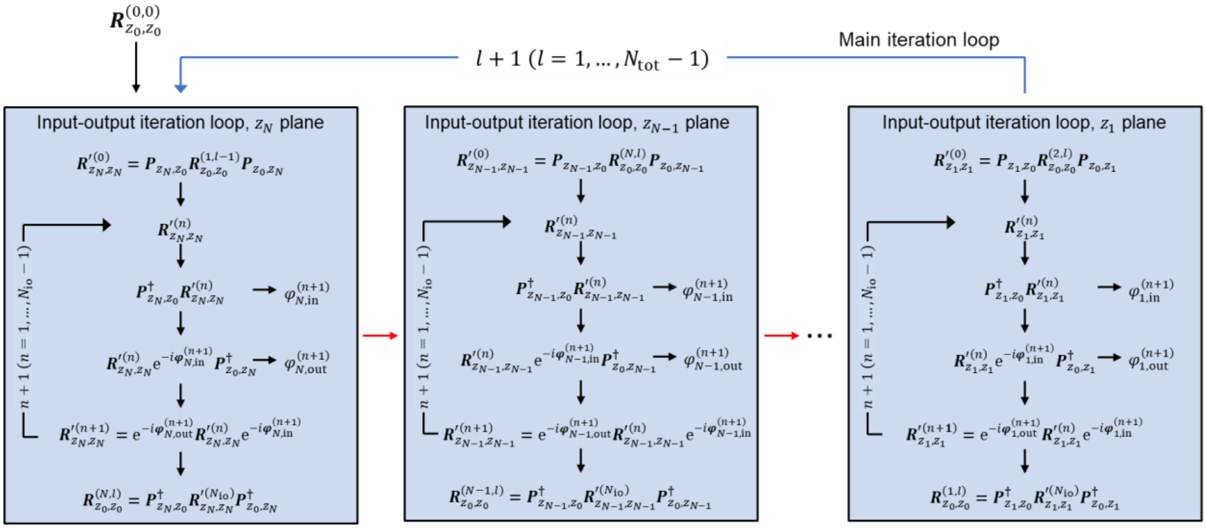

Figure S4. Flowchart of the iteration process. Each blue box corresponds to the input-output iterations of each layer from layer N to layer 1 in the lth iteration. Red arrows indicate the iteration to the next layers within the lth iteration. The blue arrow indicates the next round of iteration to the $(l+1)^{\mathrm{th}}$ iteration.

2.3 Sampling area of the phase functions

MST algorithm iteratively finds the phase functions φ_k describing the transmission matrix of the scattering medium. In this process, we need to numerically propagate the measured reflection matrix $\boldsymbol{R}_{z_0,z_0}$ to a layer position z_k by multiplying a propagation matrix $\boldsymbol{P}_{z_0,z_k}$. The diameter L_k of the phase function φ_k that we can identify is finite due to the imaging geometry shown in Fig. S5a. From Eq. (S2), we calculate the propagated field $E(\boldsymbol{\rho}, z_k)$ when the initial electric field $E(\boldsymbol{\rho}, z_0)$ is given by a band-limited point spread function (PSF) of illumination. We can get ensemble-averaged field amplitude distribution at z_k plane from a given ROI, $L_0 \times L_0$, at the z_0 plane. Figure S5b shows an ensemble-averaged amplitude distribution on an exemplary condition: wavelength $\lambda = 1.3$ μm, medium refractive index $n_0 = 1.33$, $NA = 0.9$, $z_k = 300$ μm, and $L_0 = 100$ μm. From the full width half maximum (FWHM) of the amplitude distribution, we can determine the required diameter L_k as a function of the NA and the propagation length z_k (Fig. S5c). As expected, $L_k - L_0$ is proportional to z_k. Its slope increases with NA. Under our experimental condition of $NA = 0.9$, the slope was approximately 1.2. Therefore, the L_k for our experimental system is given as $L_k \approx 1.2z_k + L_0$.

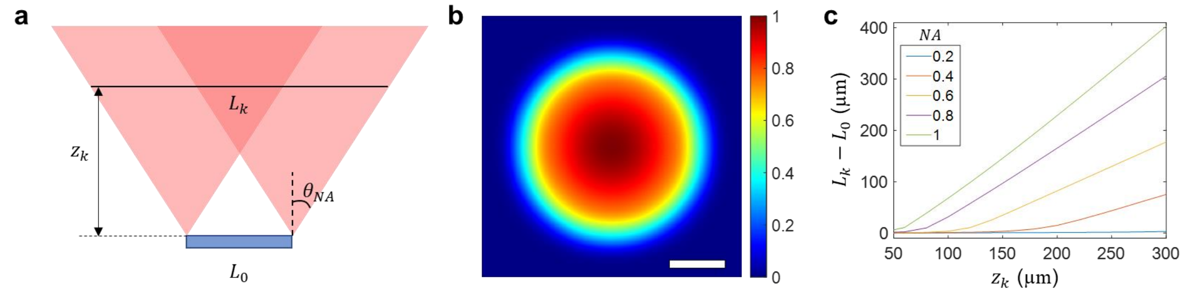

Figure S5. The diameter L_k of the sampling area for the phase functions. a, Schematic diagram illustrating an imaging cone geometry. **b**, Amplitude distribution of the ensemble-averaged propagated field for $z_k = 300$ μm. Scale bar 150 μm. **c**, L_k as a function of z_k and NA.

2.4 Spatial resolution of identifying phase function φ_k

As discussed in Sec. 2.2, we find the phase function by calculating the inner product of the columns of $\boldsymbol{R}_{z_0,z_k}$ after normalizing with the green function G. The lateral spatial resolution $\delta\rho_k$ of the phase

function is then determined by the distance z_k and the size of ROI, $L_0 \times L_0$. It can be determined by a wave focusing on a point $(\boldsymbol{\rho} = \mathbf{0}, z_k)$ from the ROI, as illustrated in Fig. S6a. In this case, the electric field emerging from the z_0 plane should be the complex conjugate of the Green function multiplied by a square aperture: $E(\boldsymbol{\rho}, z_0) = G^*(\boldsymbol{\rho}, z_k)A(\boldsymbol{\rho})$. Here the aperture function $A(\boldsymbol{\rho})$ is a 2D square function with the size of $L_0 \times L_0$, and its value is unity when $\boldsymbol{\rho}$ is within the ROI and zero otherwise. Then, the focused electric field at $(\boldsymbol{\rho}, z)$ is given as,

$$E(\boldsymbol{\rho}, z) = \int G(\boldsymbol{\rho} - \boldsymbol{\rho}'; z) \cdot G^*(\boldsymbol{\rho}', z_k)A(\boldsymbol{\rho}')\mathrm{d}^2\boldsymbol{\rho}'. \quad \text{(S18)}$$

We numerically evaluate Eq. (S18) and then calculate its FWHM in the lateral and axial directions, respectively (Figs. S6b, and S6c). The lateral resolution $\delta\rho_k$ shown in Fig. S6b is proportional to the propagation distance z_k and inversely proportional to the size of ROI L_0: $\delta\rho_k \propto z_k/L_0$. In fact, it is proportional to the inverse of the effective numerical aperture: $L_0/(2z_k)$. Note that there exists a lower bound specified by a black dashed line in the figure set by the diffraction-limited resolution of measuring $\boldsymbol{R}_{z_0,z_0}$. Similarly, δz_k also increases inversely with respect to the square of the effective numerical aperture, $(L_0/(2z_k))^2$(Fig. S6c).

Once we get the spatial resolution $\delta\rho_k$ of the layer in z_k plane, we can determine the required number of sampling points N_k of the layer by $N_k = (\pi/4)(L_k/\delta\rho_k)^2$. Since both L_k and $\delta\rho_k$ are proportional to z_k, N_k is independent of z_k. In our experimental condition, we found that $N_k \sim N_0^2 = (L_0/\Delta x)^2$ with Δx the diffraction-limited resolution. Therefore, even if the sampling area of z_k is increased, the size of $\boldsymbol{P}_{z_0,z_k}$ remains identical to the reflection matrix $\boldsymbol{R}_{z_0,z_0}$. In addition, from the axial resolution δz_k, we can determine the maximum number of layers N for describing the scattering medium. Note that the multiple scattering components caused by a smaller scatterer than the spatial resolution $\delta\rho_k$ and δz_k are outside the scope of the MST algorithm. As a result, there exist residual multiple scattering components after rectifying the quantified multiple scattering by the MST algorithm.

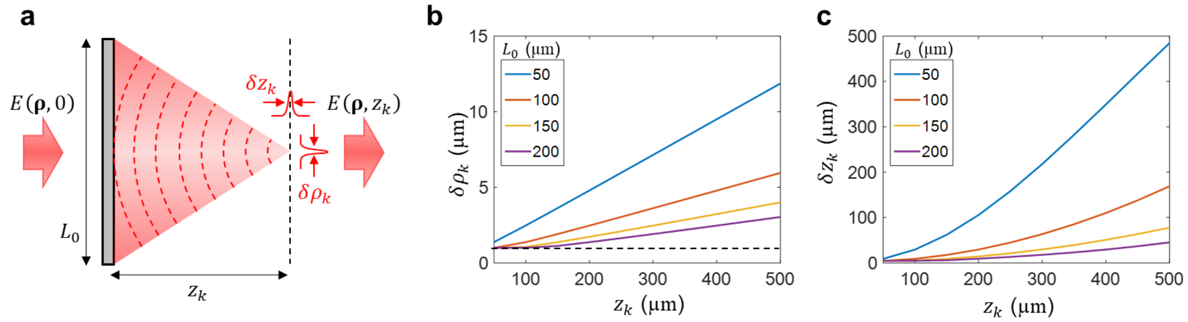

Figure S6. Spatial resolution for identifying the phase function φ_k. a, Conceptual diagram for generating a focus at $(\mathbf{0}, z_k)$ from the measured ROI, $L_0 \times L_0$. **b-c**, Lateral and axial resolutions, respectively, as a function of z_k and L_0. Black dashed line in **b**: lower bound of lateral resolution (diffraction-limited resolution of the system).

2.5 Computational cost

The spatial resolution of quantifying φ_k is determined by the size of ROI. In other words, we can trace more multiple scattering by increasing L_0. However, the computational cost for the MST algorithm increases drastically with L_0 such that there exists a practical limit of L_0. The computational complexity of the MST algorithm is mainly determined by the number of elements of the reflection matrix $\boldsymbol{R}_{z_0,z_0}$ and the propagation matrix $\boldsymbol{P}_{z_0,z_k}$. In the flow chart of the MST algorithm shown in Fig. S4, the most time-consuming processes are the matrix multiplications between $\boldsymbol{R}_{z_0,z_0}$ and $\boldsymbol{P}_{z_0,z_k}$. The size of the reflection matrix $\boldsymbol{R}_{z_0,z_0}$ for a square-shaped ROI with $L_0 = N_0\Delta x$ is $N_0^2 \times N_0^2$. As discussed in Sec. 2.3, $\boldsymbol{P}_{z_0,z_k}$ has the same dimension as $\boldsymbol{R}_{z_0,z_0}$. Therefore, the computational complexity of matrix multiplication $\boldsymbol{R}_{z_0,z_0}\boldsymbol{P}_{z_0,z_k}$ becomes $\sim\mathcal{O}(N_0^6)$. Figure S7a shows the computation time for the multiplication of two $N_0^2 \times N_0^2$ matrices by Matlab using CPU (Intel, I9 11900K, 128 Giga bytes (GB) memory) and GPU (Nvidia, RTX A6000, 48 GB memory), respectively. The computation time increases with $\sim N_0^6$, and processing with GPU is about 20 times faster. Meanwhile, the required

memory increase with N_0^4 (Fig. S7b). From Fig. S7, we can estimate the required time and memory of the MST algorithm. For example, $N_0 = 100$, the number of layers $N = 4$, $N_{\text{io}} = 10$, and $N_{\text{tot}} = 10$, which is the case in Fig. 3 in the main text, matrix multiplication takes 0.45 s, and the total number of required matrix multiplication is $2(N_{\text{io}} + 2)NN_{\text{tot}} = 960$, resulting in a total computation time of the MST algorithm about 500 seconds. For the case of experiments in Figs. 4 and 5, $N_0 = 160$ (8.5 sec. for matrix multiplication), and $N = 5$, $N_{\text{io}} = 10$, and $N_{\text{tot}} = 10$, resulting in a total computation time of about 3.4 hours.

From the discussion in Sec. 2.4, the spatial resolution of quantifying φ_k is proportional to $1/L_0$. This means that we can trace more multiple scattering with larger size of ROI. However, the computational cost increase with L_0^6, and the required memory size increases with L_0^4. This sets a practical limit for increasing L_0. For example, when $L_0 = 200$ µm, and $N_0 = 280$, the required memory size becomes approximately 128 GB. The GPU with this size of memory is not available, and CPU takes 1.2 hour for each matrix multiplication. Since we need hundreds of matrix multiplications in our MST algorithm, it is unrealistic to work with such a large ROI. If we limit the size of ROI to a degree that the GPU memory is available, and the computation time for each matrix multiplication does not exceed 10 s for the practical implementation of the MST algorithm, the ROI should be less than 120 µm with $N_0 < 165$, which is the case of our experiments.

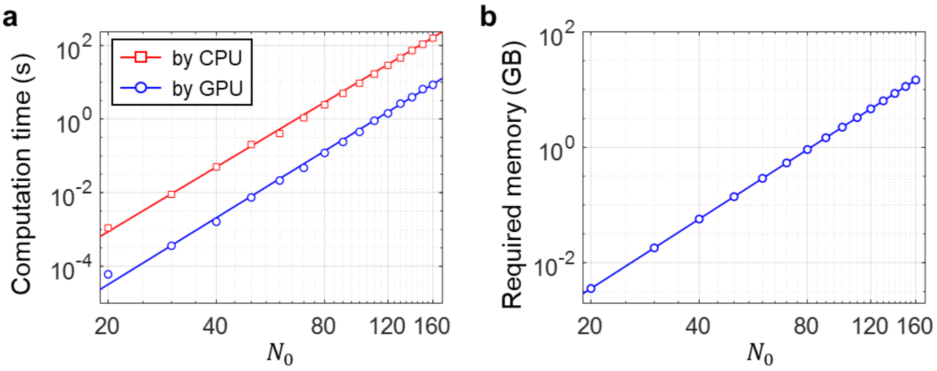

Figure S7. Computational cost for the matrix multiplication. a, Computation time for multiplying two $N_0^2 \times N_0^2$ matrices using CPU and GPU. **b,** Required computer or GPU memory for the matrix multiplication.

3. Numerical study

3.1 Detailed iteration process of the MST algorithm

In Fig. 3 of the main text, we presented the demonstration of the MST algorithm using numerically generated data. We numerically prepared ground-truth phase functions φ_k and the corresponding reflection matrix $\boldsymbol{R}_{z_0,z_0}$ based on Eqs. (S8)-(S9). Then, we applied the MST algorithm, as shown in Fig. S8. In each main iteration step, we calculated the intensity enhancement of the MST image with respect to the initial image shown in Fig. 3d of the main text (Fig. S8a). In addition, we calculated the Pearson correlation of the MST image and the reconstructed transmission matrix $\boldsymbol{T}^{\text{c}}$ with respect to their ground truths (Fig. S8b). Both the signal enhancement and correlation were increased with iteration and saturated when the iteration number was greater than 6. In Fig. S8c, we display the detailed iteration process. The individual gray panels display the process of the main iteration loop for iteration numbers, $l = 1, 2,$ and 6. In each gray panel, we illustrate the process of the input-output iteration loop of four layers starting from the fourth layer to the first layer. We present two representative complex maps of the column vectors of $\boldsymbol{R}_{z_0,z_4}$ for row indices i and j after normalizing the Green function in Eq. (S3). By Eq. (S15), the correlation angle between the two complex maps provides the relative phase angle of the corresponding position $\boldsymbol{\rho}_i$ and $\boldsymbol{\rho}_j$. With the progress of the iterations, the amplitude contrast increases while the phase noise decreases because the residual multiple scattering components M'' in Eq. (S15) decreases. On the right-hand side of each gray panel, we present the MST image and the phase functions $\varphi_k^{(l)}$ of the corresponding iteration step. With the progress of the main iteration

loop, both the phase functions and the MST image converge to the ground-truths displayed in Fig. 3 of the main text.

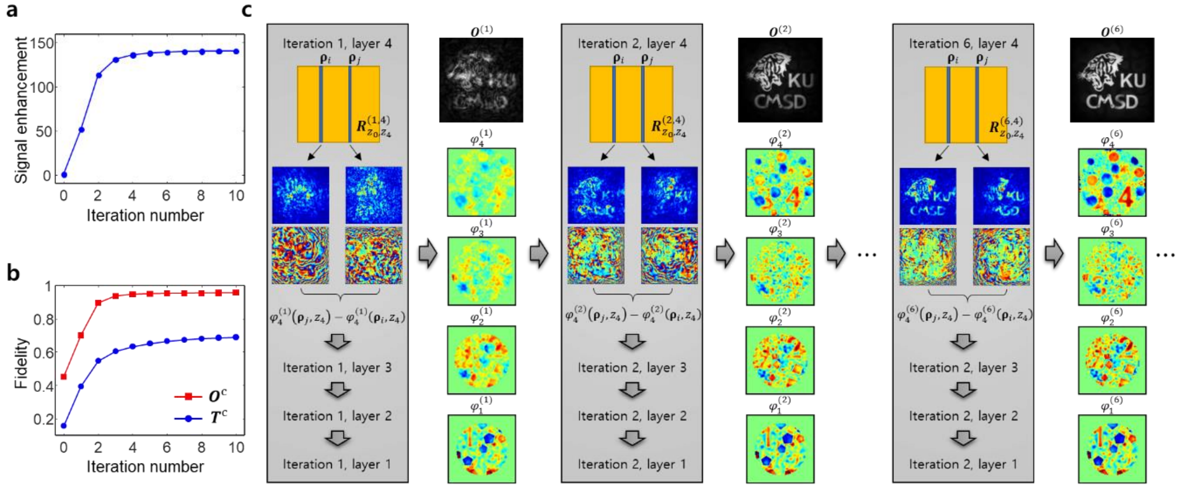

Figure S8. Detailed iteration procedure for the numerical simulation in Fig. 3. a, Intensity enhancement of the MST image with iteration. **b**, Fidelity as a function of iteration number obtained by calculating the Pearson correlation coefficients of the MST image and $\boldsymbol{T}^{\mathrm{c}}$ with respect to their ground truths. **c**, Detailed iteration process. Individual gray panels illustrate the input-output iterations of all four layers for quantifying $\varphi_k^{(l)}$ for the iteration numbers of $l =$ 1, 2, and 6 from the left. Two representative complex field maps are shown for the two column vectors of $\boldsymbol{R}_{z_0,z_4}$ at $\boldsymbol{\rho}_i$ and $\boldsymbol{\rho}_j$ after normalizing the Green function. Upper and lower images show amplitude and phase maps, respectively. On the right-hand side of each gray panel, we displayed the resulting MST image and phase map of $\varphi_k^{(l)}$.

3.2 Single- and multiple-scattered waves before and after applying the MST algorithm

In the main text, we validated the MST algorithm with a numerical simulation. In this section, we compared the original reflection matrix $\boldsymbol{R}_{z_0,z_0}$ and rectified reflection matrix $\boldsymbol{R}^{\mathrm{c}}_{z_0,z_0}$ obtained after applying the MST algorithm. As shown in Fig. S9a, there are strong off-diagonal components in the reflection matrix $\boldsymbol{R}_{z_0,z_0}$ induced by the scattering layers. After quantifying the phase functions φ_k of each layer, we reconstruct the transmission matrix $\boldsymbol{T}^{\mathrm{c}}$ from Eq. (S1). As shown in Fig. S9b, it also has strong off-diagonal components generated by the quantified multiple scattering trajectories. Then, the multiple scattering in $\boldsymbol{R}_{z_0,z_0}$ is rectified to $\boldsymbol{R}^{\mathrm{c}}_{z_0,z_0}$ by applying the inverse of $\boldsymbol{T}^{\mathrm{c}}$. After the rectification, only the diagonal part of $\boldsymbol{R}^{\mathrm{c}}_{z_0,z_0}$ becomes bright and distinct (Fig. S9c). This supports that the identified multiple scattering trajectory by the phase functions φ_k are successfully converted to the single-scattered waves (SW). Here, SW is the wave that travels ballistically through the scattering layers and scatters only one time by the target object.

We can also visualize the effect of the MST algorithm by investigating the change in the average point spread function. As described in Sec. 1.2, individual columns of $\boldsymbol{R}_{z_0,z_0}$ and $\boldsymbol{R}^{\mathrm{c}}_{z_0,z_0}$ are vectorized output PSF under a focused illumination. By shifting back each output PSF by an amount of illumination position, we obtain the average intensity of output PSF (Figs. S9d-e). Before applying the MST algorithm (Fig. S9d), we observe both a sharp peak in the center corresponding to SW and a broad background by multiple scattering. On the contrary, after applying the MST algorithm (Fig. S9e), the PSF becomes single-peaked with enhanced peak intensity. From the cross-section profiles of these PSFs (Fig. S9f), the contribution of SW and multiple scattering before the MST correction can be obtained by a double Gaussian fitting (black dashed curve). From the fitting, we measured that the height of the narrow SW peak was about $5\times10^{-3} \pm 2.4\times10^{-4}$ while that of the multiple scattering background was about $3\times10^{-3} \pm 4.6\times10^{-5}$. This means that the ratio between SW and multiple scattering passing through the confocal gating was about 1.67. In addition, the full width half maximum (FWHM) of the SW peak was about 0.44 μm, in agreement with the diffraction-limited resolution $\lambda/2$ of the system. The FWHM of the multiple scattering background was about 13.7 μm, which was about 31 times broadened due to the multiple scattering. After the MST correction, the peak height of the PSF was increased about

125 times with respect to the initial PSF with near-diffraction-limited FWHM of 0.52 μm. This substantial increase is due to the conversion of broad multiple scattering background to SW, which is evident from the fact that the broad multiple scattering background (red curve) to almost removed after the MST correction (blue curve). All these results indicate that multiple scattering trajectories were successfully rectified.

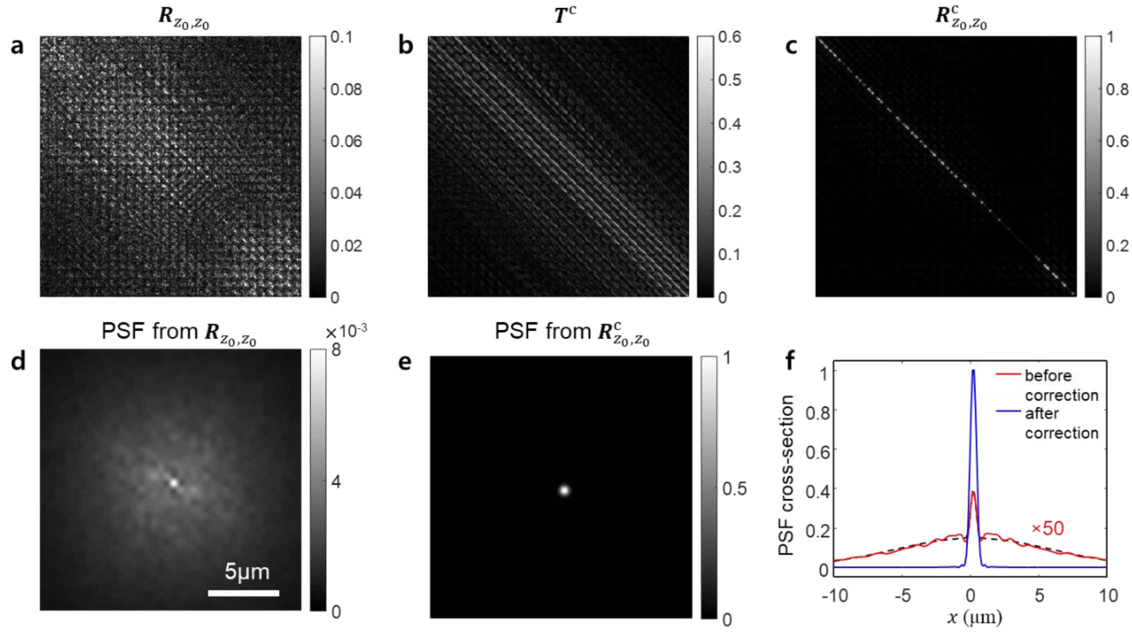

Figure S9. Comparison between the reflection matrix R_{z_0,z_0} and the rectified matrix $R^c_{z_0,z_0}$. a, Amplitude map of the reflection matrix R_{z_0,z_0}. For better visualization, we only present $20 \times 20\ \mu m^2$ ROI in the central part of R_{z_0,z_0}. **b**, Amplitude map of the identified transmission matrix T^c for the same region in **a**. **c**, Rectified matrix $R^c_{z_0,z_0}$ by T^c. Color scales of **a-c** are normalized by the maximum value of **c**. **d-e**, PSFs obtained from **a** and **c**, respectively. **f**, Cross-section profiles of the PSFs in **d-e** along the horizontal axis. The cross-section profile of **d** was multiplied by a factor of 50 (red curve) for better visualization. Black dashed curve: double Gaussian fit of the red solid curve.

3.3 Numerical demonstration of MST algorithm with randomly patterned layers

In the main manuscript, we introduced artificial polygonal and number shapes in the phase patterns in Fig. 3, for the ease of visual verification of the MST algorithm's effectiveness. In reality, we overlaid these artificial shapes with random speckle patterns, as depicted in Fig. 3b of the main manuscript, to mimic a realistic scattering medium. While not immediately apparent, the MST algorithm also managed to accurately recover the superimposed random patterns.

To further emphasize that our algorithm performs efficiently with random phase patterns, which is the case of general tissues, we conducted an additional numerical simulation. This simulation was executed with all phase plates filled exclusively with random phase patterns. As demonstrated in Fig. S10, the MST algorithm successfully reconstructed these general random phase structures with high fidelity. We quantified the accuracy of the MST algorithm's performance by computing the Pearson correlation coefficients (PCC) between the reconstructed phase map and the corresponding ground-truth values. The obtained correlation coefficients for the four layers were as follows: 0.87, 0.62, 0.64, and 0.71. Additionally, the PCC value for the reconstructed image of the target object and its ground-truth reached 0.94 with additional suppression of residual multiple scattering noise using confocal gating. This analysis confirmed the algorithm's robustness in reconstructing random phase structures accurately.

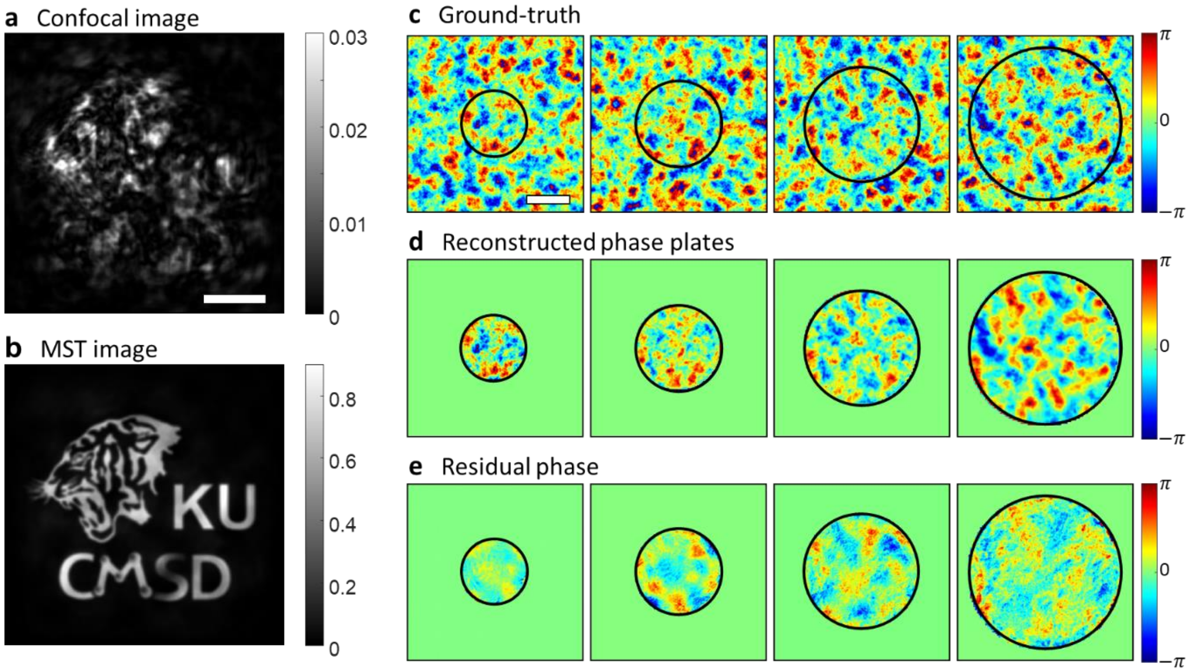

Figure S10. Numerical demonstration of MST algorithm with randomly patterned layers. a-b, Intensity map of confocal and MST images of the target, respectively, reconstructed from numerically generated data. Scale bar, 10 μm. Color scales for both images are normalized by the maximum intensity of **b**. **c**, Ground-truth phase map of phase plates used in the numerical simulation. Scale bar, 50 μm. **d-e**, Reconstructed phase maps from MST algorithm, and residual phase maps with respect to the ground-truth phase maps in **c**, respectively. Circles correspond to the reconstruction area in the MST algorithm whose diameter depends on the distance of the layer from the target object.

4. Further experimental studies

4.1 Behavior of multiple scattering rectification by MST algorithm in *in-vivo* imaging

In this section, we assess the performance of the MST algorithm for *in vivo* image data in Fig. 6. As shown in Fig. S11a, we applied the MST algorithm to the reflection matrix measured at 270 μm depth from the surface of the skull with the increase of the number of phase plates N. Like Fig. 5e in the main text, the image contrast was increased with N. We obtain the point spread function (PSF) of $\boldsymbol{R}^{\mathrm{c}}_{z_0,z_0}$ in Fig. S11b by averaging the intensity of each column images in de-scanned coordinates (see section 3.2 for details). Unlike the PSF presented in Fig. S9d, the initial PSF spreads over the whole ROI without revealing the ballistic peak in the center due to severe multiple scattering and low target reflectance. By applying the MST algorithm, the background of the PSF is reduced while the central ballistic peak is increased. The remaining background in the PSF corresponds to the remaining multiple scattering components that could not be rectified with the MST algorithm. Due to the spatial resolution of the phase plate reconstruction discussed in section 2.4, and the discrete layer model approximation, not all the multiple scattering trajectories can be traced with the MST algorithm. It is noteworthy that the level of the background with respect to the ballistic peak is decreased with N, indicating that more multiple scattering backgrounds were converted into the ballistic signal.

For the quantitative assessment of the multiple scattering rectification by the MST, we obtain the cross-section of the PSF with N (Fig. S11c). We fit the cross-section of the PSF with the following function,

$$f_{\mathrm{PSF}}(x) = I_{\mathrm{b}} \exp\left[-(x - x_0)^2/w_{\mathrm{b}}^2\right] + I_{\mathrm{M}} \exp\left[-(x - x_0)^2/w_{\mathrm{M}}^2\right], \quad \text{(S19)}$$

which is the summation of a narrow ballistic peak and broad multiple scattering background. Here I_{b}, and I_{M} are the peak intensities of the ballistic signal and multiple scattering, respectively, at the center at $x = x_0$, where the confocal gate is located for the image formation. And w_{b} and w_{M} correspond to the width of the ballistic peak and the multiple scattering background, respectively. First, we investigated the ratio of the ballistic peak I_{b} with respect to the total signal $I_{\mathrm{b}} + I_{\mathrm{M}}$. Before applying

MST algorithm, we could not identify this ratio because the ballistic signal was completely obscured by the severe multiple scattering. This explains that no structures were resolved in the conventional confocal reflectance image shown in the first column of Fig. S11a. After applying the MST algorithm, the ratio of ballistic peak $I_\mathrm{b}/(I_\mathrm{b}+I_\mathrm{M})$ shown as red square dots in Fig. S11d increase up to 0.85 with N. This means that the contribution of multiple scattering in the MST images of Fig. S11a is reduced to less than 15% of the total confocal signal by rectifying the quantified multiple scattering trajectories. Next, we calculated the contribution of the remaining multiple scattering signal (the second term in Eq. (S19)) from the fitted function with respect to the total multiple scattering signal (Fig. S11d). The total multiple scattering was estimated by the initial PSF, where the ballistic signal was negligible. For the case of $N = 1$ (single phase plate model), the contribution of the remaining multiple scattering signal was about 0.93, which means that the MST algorithm rectified about 7% of the multiple scattering trajectories. With the increase of N, this value decreased to 0.67, confirming that we could rectify about 33% of multiple scattering into the ballistic signal.

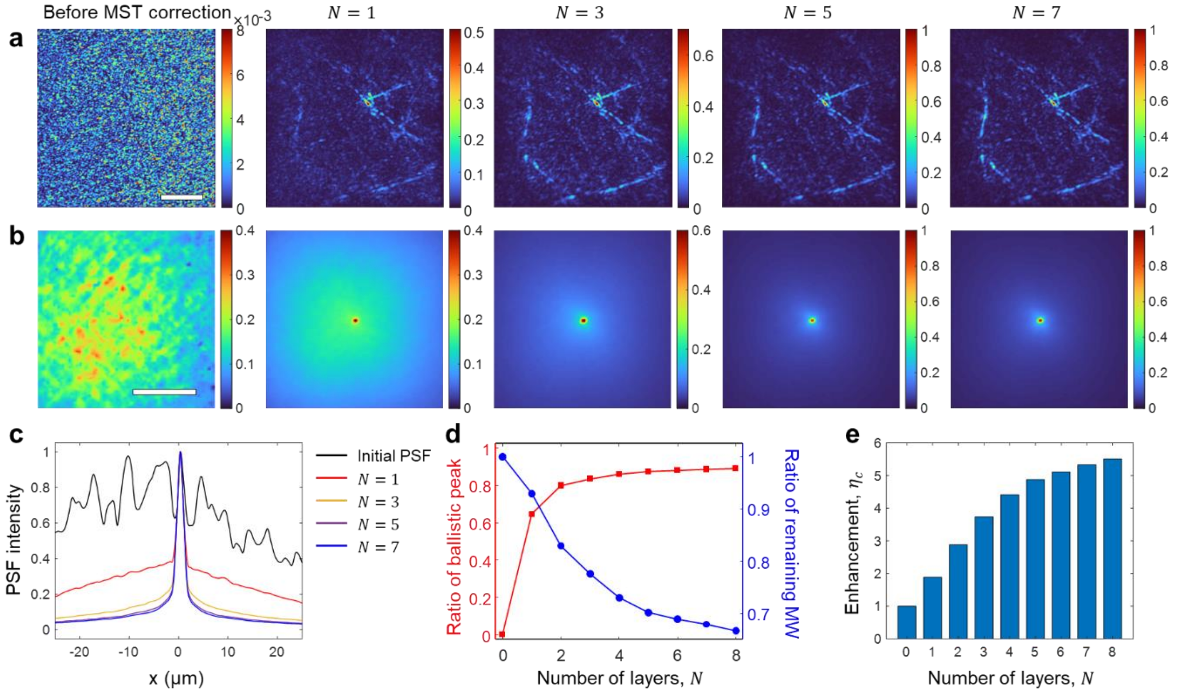

Figure S11. Performance of the MST algorithm in the *in-vivo* imaging depending on the number of phase plates N. a, Confocal reflectance image and MST images of the mouse brain at 270 μm depth with the increase of N. Color bar: normalized by the maximum intensity of the MST image with $N = 7$. Scale bar: 20 μm. **b**, Average point spread function obtained by $\boldsymbol{R}^{\mathrm{c}}_{z_0,z_0}$ with N. Color bar: normalized by the maximum intensity of the PSF for $N = 7$ case. Scale bar: 20 μm. **c**, Cross-section profiles of PSFs along the x-axis after normalizing by its peak intensity. **d**, The ratio of the ballistic peak with the total signal at the confocal spot (red square dots), and the ratio of the remaining multiple scattering intensity with respect to the initial total multiple scattering after applying MST algorithm. **e**, Intensity enhancement of MST images as a function of N.

4.2 Comparison with existing imaging modalities

The proposed MST method enables deep imaging inside a thick scattering tissue. In Fig. 6, we compared the performance of the MST method with the conventional confocal microscopy for *in vivo* mouse imaging. Here, we compared the MST image with more advanced conventional imaging modalities based on the reflection matrix. In Fig. S12b, we applied the CLASS algorithm to the measured reflection matrix for correcting the specimen-induced aberrations [7]. However, due to the severe scattering and the aberrations, it didn't work properly, and we could not identify any myelinated axons. In this case, aberration correction by the CLASS algorithm failed because the shift invariance assumed by the CLASS algorithm is no longer valid due to position-dependent aberrations. Next, we applied the CLASS algorithm by dividing the ROI into 8×8 patches to address the position-dependent

aberrations [1]. As shown in Fig. S12c, multiple scattering background is greatly suppressed, and we could identify some pieces of the myelinated axons. However, it shows poor image quality, showing fragmented myelin because CLASS works well for some patch areas but not for others. In addition, we also applied the previous state-of-the-art conjugate adaptive optics approach – the so-called conjugate CLASS [2] – to the measured reflection matrix (Fig. S12d). It shows improved image quality with myelinated axons well-connected, but some myelin segments were missing due to the strong multiple scattering background compared with the MST image in Fig. S12e.

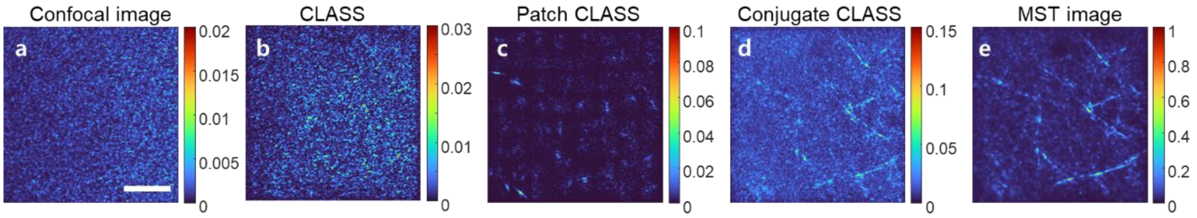

Figure S12. Comparison with other imaging modalities. a, Confocal reflectance image. Scale bar: 30 μm. **b-d**, CLASS image, CLASS image with patches, and conjugate-CLASS image, respectively. **e**, MST image. Color bars of all the images are normalized by the maximum intensity of the MST image in **e**.

5. Quantitative analysis of the performance of the MST algorithm

In the numerical study in Sec. 3.1, we validated the accuracy of the MST algorithm by measuring the Pearson correlation between the reconstructed object image and its ground truth. In the case of experimental data, there is no knowledge of the ground truth. Therefore, we need other metrics for validating the performance of the algorithm. This is necessary for tuning the configuration parameters such as the position of the layers, the number of layers, the maximum iteration number, the tolerance of the iteration, and so on. Here, we propose two criteria that represent the performance of our algorithm in the absence of a ground-truth image: (1) the enhancement of SW by inversing $\boldsymbol{T}^{\mathrm{c}}$ for the illumination and the detection paths, and (2) the enhancement of SW after applying the MST algorithm.

In the first criterion, we evaluate the performance of the MST algorithm by the ballistic attenuation coefficient of the reconstructed transmission matrix $\boldsymbol{T}^{\mathrm{c}}$. If we consider a scattering medium whose thickness is d and the scattering mean free path (SMP) is l_s, the average intensity of the ballistic wave (BW) will decay by the Beer-Lambert's law as, $I_B \propto \mathrm{e}^{-d/l_{\mathrm{s}}}$. If the transmission matrix of the medium is given by $\widetilde{\boldsymbol{T}}^{\mathrm{c}}$ in the spatial frequency domain, we can calculate the intensity ratio of BW by the summation of the diagonal element of $\widetilde{\boldsymbol{T}}^{\mathrm{c}}$ with respect to the total intensity. Therefore, we can calculate the ballistic attenuation coefficient $\mathrm{e}^{-d/l_{\mathrm{s}}}$ of $\boldsymbol{T}^{\mathrm{c}}$ by

$$\eta_{\mathrm{T}} = \sum_i \left|\tilde{T}_{ii}^{\mathrm{c}}\right|^2 \Big/ \sum\sum_{i,j} \left|\tilde{T}_{ij}^{\mathrm{c}}\right|^2, \tag{S20}$$

where $\tilde{T}_{ij}^{\mathrm{c}}$ is the matrix element of $\widetilde{\boldsymbol{T}}^{\mathrm{c}}$ in spatial frequency domain obtained by the Fourier transform of $\boldsymbol{T}^{\mathrm{c}}$. In the MST algorithm, we rectify multiple scattering by multiplying the inverse of $\boldsymbol{T}^{\mathrm{c}}$ as if there exist virtual inverse scattering layers in front of the scattering medium. As a result, the intensity of BW after the MST correction will increase by a factor of $1/\eta_{\mathrm{T}}$. The intensity of SW after the round-trip reflection will increase by a factor of $1/\eta_{\mathrm{T}}^2$. Thus, we can set the metric for the first criterion as $\xi_1 = 1/\eta_{\mathrm{T}}^2$. For example, for the case of the numerical simulation in Fig. 3, $\eta_{\mathrm{T}} = 5.4 \times 10^{-2}$, and $\xi_1 = 344$ were obtained by the MST algorithm. This means that multiple scattering was converted to SW such that SW intensity was increased by 344 times. The attenuation factor $\mathrm{e}^{-d/l_{\mathrm{s}}}$ of the ground-truth transmission matrix $\boldsymbol{T}$ was $\mathrm{e}^{-3.22}$ while that of the reconstructed $\boldsymbol{T}^{\mathrm{c}}$ and the corrected matrix $\boldsymbol{T}(\boldsymbol{T}^{\mathrm{c}})^{\dagger}$ by the MST algorithm were $\mathrm{e}^{-2.92}$, and $\mathrm{e}^{-0.424}$, respectively.

In the second criterion, we compare the contribution of SW in $\boldsymbol{R}_{z_0,z_0}$ and $\boldsymbol{R}_{z_0,z_0}^{\mathrm{c}}$ from the intensities of their diagonal elements, which correspond to the signal passing through the confocal gating:

$$\eta_{\mathrm{c}}=\frac{\sum_i |R_{ii}^{\mathrm{c}}|^2}{\sum_i |R_{ii}|^2}. \tag{S21}$$

Here R_{ii}, and R_{ii}^{c} are diagonal elements of $\boldsymbol{R}_{z_0,z_0}$ and $\boldsymbol{R}_{z_0,z_0}^{\mathrm{c}}$, respectively. At first glance, the ratio η_{c} can be a good metric for describing the performance of the MST method since it describes the enhancement of SW. However, this statement can fail when the initial multiple scattering is too strong. The diagonal elements of $\boldsymbol{R}_{z_0,z_0}$ cannot be considered as a pure SW, meaning that the diagonal elements of $\boldsymbol{R}_{z_0,z_0}$ don't properly represent the initial contribution of SW. For instance, the confocal image shown in Fig. 3d of the main text does not show a clear object image due to multiple scattering components that survive the confocal gating. For better estimation of SW enhancement, we introduce another parameter α, the contribution of SW in the diagonal element of $\boldsymbol{R}_{z_0,z_0}$ as, $\alpha = I_{\mathrm{S,diag}}/I_{\mathrm{diag}}$, where I_{diag} is the total intensity of the diagonal elements, while $I_{\mathrm{S,diag}}$ is the intensity of SW in the diagonal. As discussed in Fig. S9f, we can estimate $I_{\mathrm{S,diag}}$ from the double Gaussian fitting of the cross-section of the PSF if we can identify the SW peak from the initial PSF. Finally, we can define the metric of the second criterion as $\xi_2 = \eta_{\mathrm{c}}/\alpha$, as shown in Fig. S13. It should be noted that the estimation of α, and, thus, the ξ_2, may not be possible in the extreme case when multiple scattering is so strong that the ballistic peak is not visible, as depicted in Fig. S13i.

We summarize the parameters and metrics related to these criteria for the numerical and experimental data presented in the main text in Table S1. ξ_1 and ξ_2 were similar for the data in Figs. 3-5, but there was a substantial difference between the two for the data in Fig. 6. This is because the initial multiple scattering was too strong to even evaluate α in the case of through-skull imaging data. ξ_2 given in the table is the estimation for $\alpha = 1$, and the real ξ_2 should be much larger. In this extreme case, $\xi_1 = 122$ is a much more reliable criterion. Although the two criteria ξ_1 and ξ_2 are related to the enhancement of SW, they have a slight difference. ξ_2 is derived from the reconstructed object matrix $\boldsymbol{R}_{z_0,z_0}^{\mathrm{c}}$, and thus depends on the target object. On the contrary, ξ_1 is independent of the target object as it is derived from $\boldsymbol{T}_{\mathrm{in}}^{\mathrm{c}}$. If we consider the quality of the MST image, ξ_2 is a relevant criterion describing the performance of the MST algorithm. However, if we are more interested in the rectification of multiple scattering, ξ_1 can be more relevant.

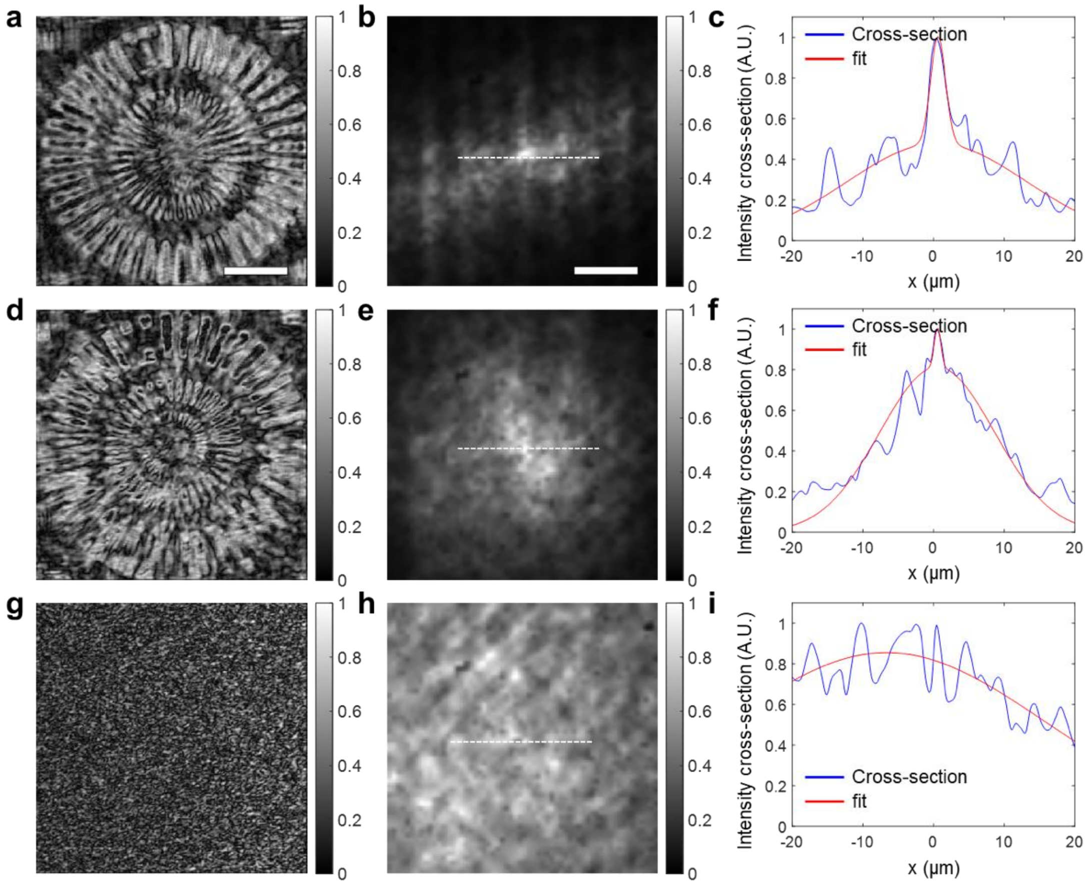

Figure S13. Determination of α from experimental data in Figs. 4-6. a-c, Confocal reflectance image, point spread function, and its cross-section along the x-axis, respectively, for the data shown in Fig. 4 (a resolution target under an onion tissue). Scale bar in **a**: 20 µm. Scale bar in **b**: 10 µm. **d-f**, Same as **a-c,** but for the data in Fig. 5 (a resolution target under a 180-µm-thick mouse skull). **g-i,** Same as **a-c,** but for the data in Fig. 6 (*in vivo* brain tissue imaging through a 200-µm-thick mouse skull).

	**Criterion 1**	**Criterion 2**
**Numerical simulation in Fig. 3**	$\eta_T = 5.4 \times 10^{-2}$<br>$d/l_s = 2.92$<br>$\xi_1 = 344$	$\eta_c = 136$<br>$\alpha = 0.63$<br>$\xi_2 = 216$
**Onion tissue data in Fig. 4**	$\eta_T = 5.4 \times 10^{-2}$<br>$d/l_s = 2.92$<br>$\xi_1 = 343$	$\eta_c = 37$<br>$\alpha = 0.56$<br>$\xi_2 = 66$
**Mouse cranial tissue data in Fig. 5**	$\eta_T = 5.3 \times 10^{-2}$<br>$d/l_s = 2.94$<br>$\xi_1 = 360$	$\eta_c = 111$<br>$\alpha = 0.19$<br>$\xi_2 = 584$
**Through-skull brain imaging in Fig. 6**	$\eta_T = 0.09$<br>$d/l_s = 2.4$<br>$\xi_1 = 122$	$\eta_c = 5.5$<br>α: unidentified<br>$\xi_2 = 5.5 \ (\alpha = 1)$

Table S1. Evaluation of the MST algorithm for various data by two criteria, ξ_1 and ξ_2. α value for Fig. 6 was unidentified due to the strong initial multiple scattering. We assumed $\alpha = 1$ for estimating ξ_2, which is a significant underestimation. Therefore, $\xi_1 = 122$ is a much more reliable criterion for data in Fig. 6.

References

1. Yoon, S., et al., *Laser scanning reflection-matrix microscopy for aberration-free imaging through intact mouse skull.* Nat Commun, 2020. **11**(1): p. 5721.
2. Kwon, Y., et al., *Computational conjugate adaptive optics for longitudinal through-skull imaging of cortical myelin.* bioRxiv, 2022: p. 2022.05.18.492378.
3. Kang, S., et al., *Imaging deep within a scattering medium using collective accumulation of single-scattered waves.* Nature Photonics, 2015. **9**(4): p. 253-258.
4. Jeong, S., et al., *Focusing of light energy inside a scattering medium by controlling the time-gated multiple light scattering.* Nature Photonics, 2018. **12**(5): p. 277-283.
5. Goodman, J., *introduction to Fourier Optics*. 1968: McGraw-Hill.
6. Nash, J.C. and S. Shlien, *Simple algorithms for the partial singular value decomposition.* The Computer Journal, 1987. **30**(3): p. 268-275.
7. Kang, S., et al., *High-resolution adaptive optical imaging within thick scattering media using closed-loop accumulation of single scattering.* Nat Commun, 2017. **8**(1): p. 2157.
